# Exploring the Formation of a Disjunctive Pattern between Eastern Asia and North America Based on Fossil Evidence from *Thuja* (Cupressaceae)

**DOI:** 10.1371/journal.pone.0138544

**Published:** 2015-09-22

**Authors:** Yi-Ming Cui, Bin Sun, Hai-Feng Wang, David Kay Ferguson, Yu-Fei Wang, Cheng-Sen Li, Jian Yang, Qing-Wen Ma

**Affiliations:** 1 State Key Laboratory of Systematic and Evolutionary Botany, Institute of Botany, Chinese Academy of Sciences, No. 20 Nan Xin Cun, Xiangshan, Beijing, 100093, China; 2 Department of Paleontology, University of Vienna, Althanstrasse 14, A-1090, Vienna, Austria; 3 Beijing Museum of Natural History, Beijing, 100050, China; 4 University of the Chinese Academy of Sciences, Beijing, 100039, China; Agharkar Research Institute, INDIA

## Abstract

*Thuja*, a genus of Cupressaceae comprising five extant species, presently occurs in both East Asia (3 species) and North America (2 species) and has a long fossil record from Paleocene to Pleistocene in the Northern Hemisphere. Two distinct hypotheses have been proposed to account for the origin and present distribution of this genus. Here we recognize and describe *T*. *sutchuenensis* Franch., a new fossil *Thuja* from the late Pliocene sediments of Zhangcun, Shanxi, North China, based on detailed comparisons with all living species and other fossil ones, integrate the global fossil records of this genus plotted in a set of paleomaps from different time intervals, which show that *Thuja* probably first appeared at high latitudes of North America in or before the Paleocene. This genus reached Greenland in the Paleocene, then arrived in eastern Asia in the Miocene via the land connection between East Asia and western North America. In the late Pliocene, it migrated into the interior of China. With the Quaternary cooling and drying, *Thuja* gradually retreated southwards to form today’s disjunctive distribution between East Asia and North America.

## Introduction

Disjunct distribution between eastern Asia and North America is a widespread phenomenon in the Northern Hemisphere. It represents one of Thorne’s 14 types of disjunct distribution [1]. In 1750 Linnaeus and his student Jonas P. Halenius discovered that the flora of eastern Asia and eastern North America displayed similarities [2, 3]. In the 19^th^ Century Asa Gray compared the flora of North America and Japan, and further described the details of the disjunctive distribution pattern in eastern Asia and North America [4–9]. Gray’s studies encouraged many subsequent scientists to study the phenomenon of disjunctive distribution in different fields such as in floristic research [10], biogeographical studies [11–13], ecological analyses [14–16], paleobiological investigations [9, 17–19], and molecular biological research [12, 20–24]. Many explanations and hypotheses exist as to how this kind of distribution originated. In order to critically evaluate the evidence, we need sufficient fossil records to explain how this distribution pattern actually developed [18, 25, 26]. In the past hundred years the large number of paleobotanical studies carried out in North America has supplied a reliable fossil record. However, in eastern Asia and more particularly in China, the limited number of studies represents a weak link, which requires to be remedied by new and unequivocal fossil evidences.

*Thuja* (Cupressaceae) comprises 5 extant species, presently distributed in eastern Asia (3 species) and North America (2 species) (data from http://esp.cr.usgs.gov/data/little/). There are abundant fossil records of foliage attributed to this genus from the late Cretaceous to Pleistocene, but only a few specimens have been found with reproductive organs.

Based on both fossil and phylogenetic studies, two opposite hypotheses have been proposed, an origin in Asia [27] and an origin in North America [28, 29]. Li & Xiang (2005) analyzed *Thuja*’s nrDNA ITS sequences, and concluded that *T*. *standishii* and *T*. *sutchuenensis* are the most closely related species, and together with *T*. *occidentalis* form a clade, while *T*. *koraiensis* and *T*. *plicata* form another one. Li & Xiang speculate that *Thuja* was widely distributed in the Northern Hemisphere, but for many reasons the distributional area contracted and became relict in eastern Asia. After that, at about 21.2 ± 14.7 Ma they reached western North America via a land connection, and in 20.30 ± 4.79 Ma crossed the Atlantic into eastern North America via the North Atlantic Landbridge, forming the present day pattern. McIver & Basinger (1989) believed that *T*. *ehrenswaerdii* and *T*. *sutchuenensis* are sister taxa, which diverged in the Paleocene. In their opinion *T*. *polaris* represents the sister species of the common ancestor of the remaining recent species. In North America climate change in the Miocene could have caused disjunction which eventually resulted in the formation of two distinct species, *T*. *plicata* and *T*. *occidentalis*. *T*. *nipponica* found in Far Eastern Russia and Japan in the Miocene may represent the ancestor of *T*. *standishii* and *T*. *koraiensis*, which would suggest that these recent species originated after the Miocene. Based on the genes cpDNA, nrDNA ITS, and *LEAFY*, *4CL*, Peng & Wang (2008) believed that *T*. *koraiensis* and *T*. *plicata* constitute a sister species pair, while *T*. *standishii*—*T*. *sutchuenensis* represents another species pair, and *T*. *occidentalis* comprises a clade on its own. They speculated that *Thuja* originated at high latitudes in North America. Some 60 Ma ago *T*. *occidentalis* separated as a single clade, at 51.1 ± 3.96 Ma two more clades arose, one of which further subdivided to form *T*. *standishii* and *T*. *sutchuenensis* at 23.7 ± 5.04 Ma, while the other one formed *T*. *koraiensis* and *T*. *plicata* at 14.7 ± 6.06 Ma.

In this paper, we report and describe a new record of *Thuja* from the Zhangcun Formation, northern China and review the geographic history and possible migration routes of the genus.

## Materials and Methods

**Fig 1:**
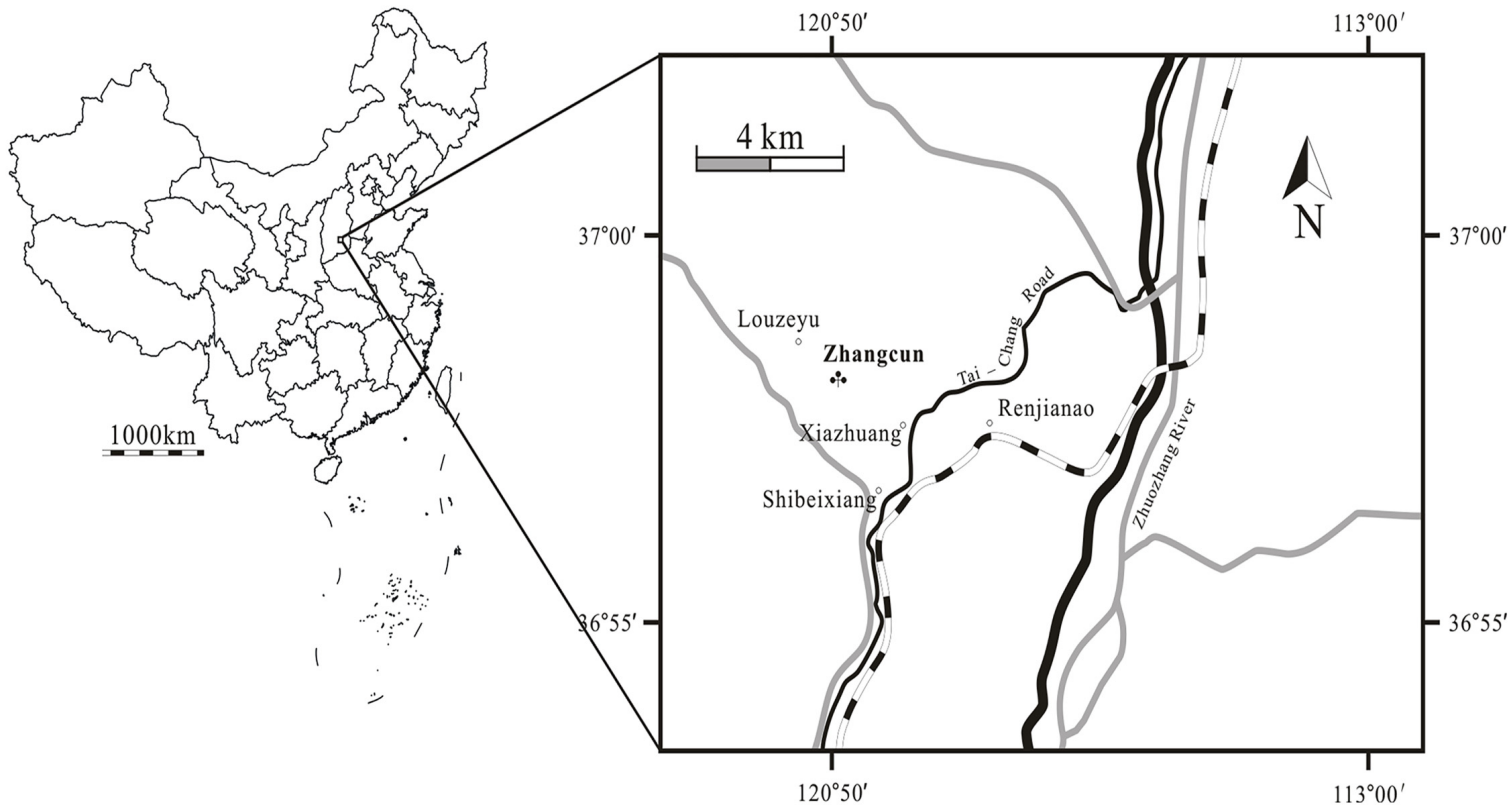
Map showing the fossil locality (♣) in Shanxi, China. The maps are created by authors using “CorelDraw 14” software.

The fossils were collected from the middle to upper part of the Zhangcun Formation, late Pliocene sediments on the southeastern margin of the Loess Plateau, Shanxi, North China (36°58′N;112°51′E, 1043 m a.s.l., Fig 1). The middle to upper part of the Zhangcun Formation is dated to 2.77–2.52 Ma BP [30].

**Fig 2:**
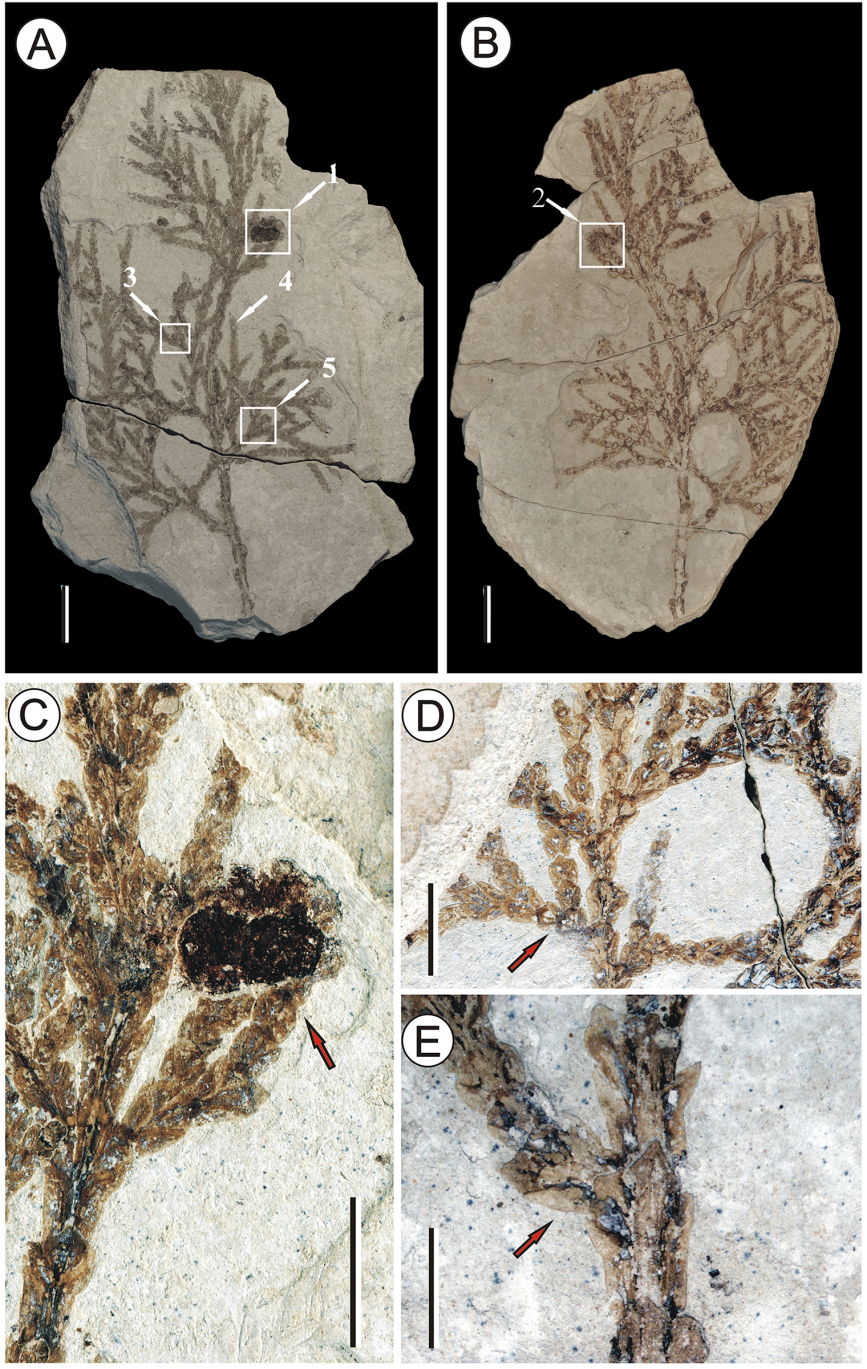
Fossil *T*. *sutchuenensis*. (A)-(B) the part and counterpart of a fertile shoot: two seed cones borne solitarily, (arrows 1 & 2) two mature seed cones, (arrows 3 & 5) basal pair of branchlet facial leaves arise in the axils of branch lateral leaves, (arrow 4) the lateral leaves not overlap with the subsequent pair, scale bar = 1 cm; (C) the arrow indicates the two mature female cones, scale bar = 5 mm; (D) the arrow shows the alternating shoots, scale bar = 5 mm; (E) the arrow indicates leaf dimorphism within main and lateral branchlets, scale bar = 3 mm.

The material consists of a compression, represented by part and counterpart (Fig 2A and 2B). The specimens examined in this article are deposited in the National Museum of Plant History of China, Institute of Botany, Chinese Academy of Sciences, No: IBCAS-SX-007, IBCAS-SX-008. The material was degaged using needles to expose the detailed characters of the foliage. Pieces of the carbon film were removed and immersed in 10% HCl for 3–4 hours, followed by 40% HF for 12 hours, washed with water, and placed in maceration fluid (50% nitric acid and a saturated solution of potassium chlorate in a ratio of 3:1) until the material became translucent. It was then washed with distilled water three times, after which the remains of the mesophyll were removed with a small brush and the cuticle dyed with safranin for 1 minute. The superfluous safranin was washed away and the cuticle embedded in glycerin and protected by a cover glass prior to examination under a light microscope. The living materials used for comparison came from the Peking Herbarium (PE) (Specimen Nos: 2070474, 1607491, 1727049, 1477906, 1345637, 1753594.). The gross-morphological terminology follows Farjon (2005) [31] and the cuticle terminology Dilcher (1974) [32]. The fossil and living materials were photographed with a Nikon D300 digital camera, and examined under a Nikon SMZ1000 stereomicroscope and a FEI Quanta 200F Scanning Electron Microscope.

We used the Point Tracker for Windows program to transform the fossil localities of *Thuja* to paleolatitudes and-longitudes, and to locate these paleolocalities on the paleomaps of Scotese (1997) [33] for the Paleocene (60 Ma) and Miocene (14 Ma). For the Pliocene and Pleistocene modern maps were utilized.

## Ethics Statement

All necessary permits were obtained for the described field studies and were granted by the local government of Shanxi Province. The field work did not involve endangered or protected species. The studies using herbarium specimen were permitted by Institute of Botany, Chinese Academy of Sciences.

## Results

**Family** Cupressaceae Gray 1821

    **Subfamily** Cupressoideae Rich. ex Sweet 1826

        **Tribe** Thujopsideae Henkel & W. Hochst 1865

            **Genus** *Thuja* Linnaeus 1753

                **Species** *Thuja sutchuenensis* Franch. 1899

### Description

Seed cones 2, terminal on short branch, broadly ovate, 4.2–4.3 mm long, 3.0–3.2 mm wide. Bract-scale complexes 4–5 pairs, decussate. Apex of bract round, and near apex there is a small umbo, the apex of umbo blunt. The second whorl of bract-scale complexes is broadly obovate, and the biggest round (Fig 2C). Branches dichotomous, and a few decussate, forming flat frond-like sprays. Branchlets flat, 4–12.9 mm long, 0.9–1.8 mm wide (Fig 2D). Leaves on branchlets scaly, adpressed, decussate, with dimorphic-flattened facial leaves and folded lateral leaves. Scale leaves on branches and branchlets display differences. On branchlets, facial leaves rhombic, 0.78–1.45 mm long, 0.65–1.21 mm wide, apex blunt, and covers the basal part of the subsequent facial leaf; lateral leaves folded, 1.5–2.5 mm long, apex blunt or acute, most of them not reaching the basal part of the subsequent lateral leaf, the apex introrse, appressed to facial leaves or slightly free. On branch, facial leaves oblong or broadly oblong, 2.9–3.8 mm long,1.24–1.97 mm wide, apex acute or blunt; lateral leaves narrowly oblong or falcate, 2.9–3.4 mm long, 0.7–1 mm wide, apex blunt or acute (Fig 2E).

**Fig 3:**
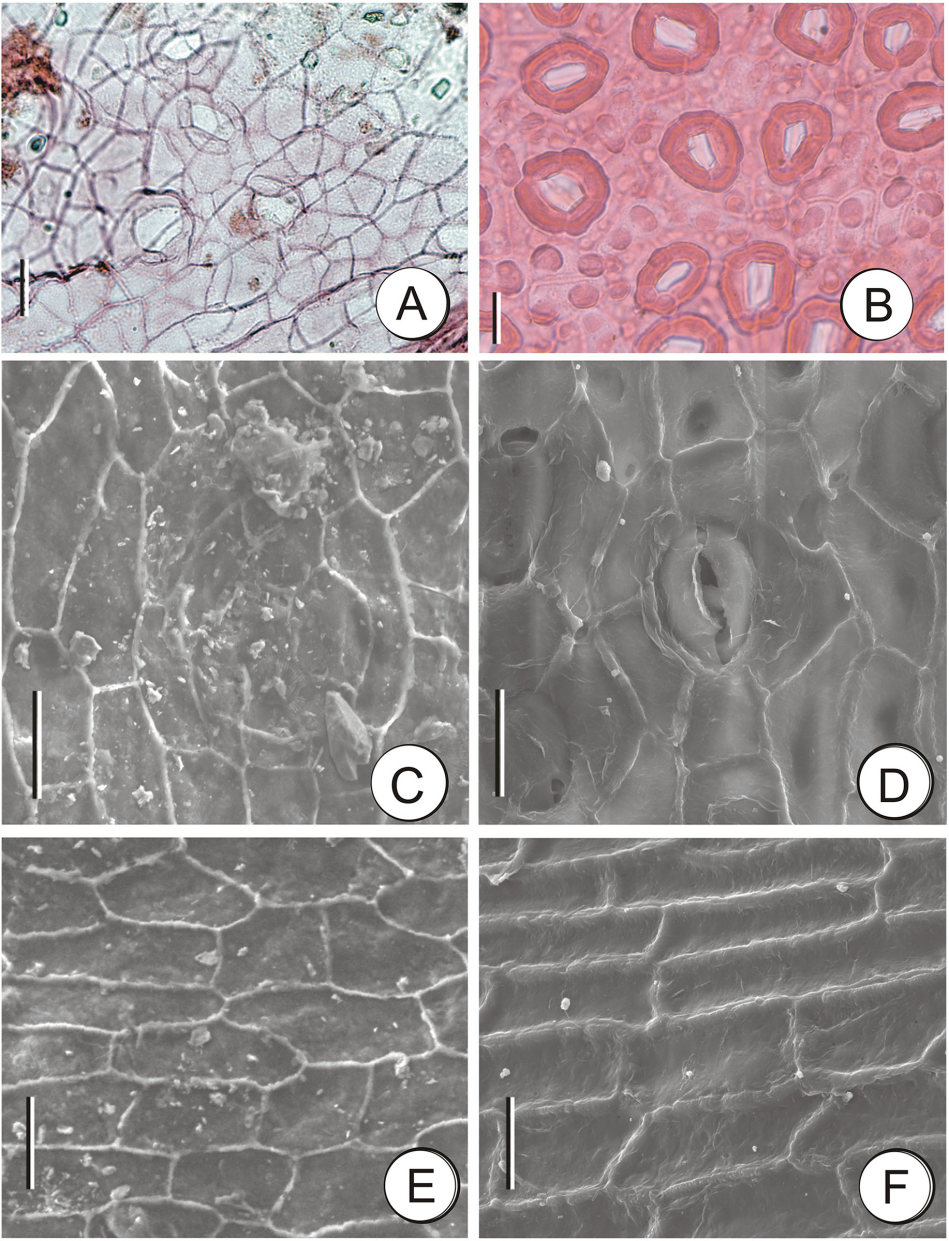
Comparison of the epidermal cell structure of fossil and living *T*. *sutchuenensis*. A, C, E, fossil *T*. *sutchuenensis* described in this article; B, D, F, living *T*. *sutchuenensis*. A, B, scale bars = 50 μm; C–F, scale bars = 20 μm.

Epidermal cells rectangular, 20×15 μm, anticlinal wall straight, continuous, margin jagged. Stomata cyclocytic, lobed Florin ring palpably on the outer surface. Stomatal subsidiary cells 5–6, 13–27×10–19 μm (Fig 3A, 3C and 3E).

### Remarks

The Zhangcun specimens demonstrate 2 female cones terminally attached on branchlets, cones broadly ovate. Bract-scale complexes 4–5 pairs, decussate, apices round. Small umbo near apex, apex blunt. Scale leaves dimorphic with appressed facial leaves and folded lateral leaves. Branchlets flattened, dichotomous, forming a flat frond-like spray. Stomata cyclocytic, with 5–6 subsidiary cells and a Florin ring. The character combination of the fossil cones and leaves falls within the circumscription of the genus *Thuja* [34].

### Comparison with Other Fossil Species of *Thuja*

Numerous fossils have been attributed to *Thuja*, but most of them represent foliage without generative organs (male and female cones) [35–46]. Because *Thuja*-like foliage is very common in the Cupressaceae (such as *Chamaecyparis*, *Platycladus* and *Calocedrus*), it is misleading to identify a foliage fossil as *Thuja*.

**Fig 4:**
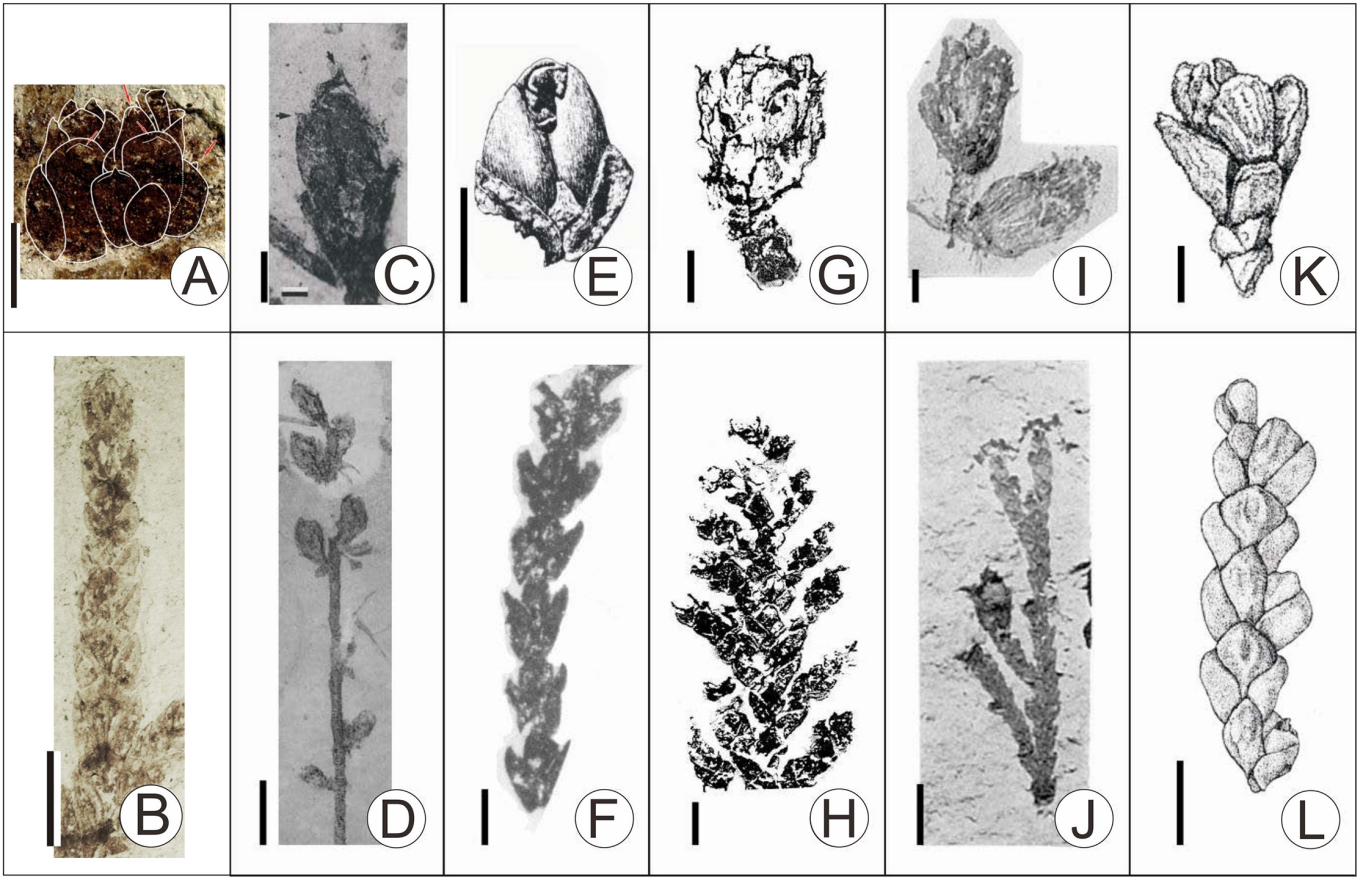
Comparison of fossil *T*. *sutchuenensis* with other fossils of *Thuja* bearing female cones. A, B, fossil *T*. *sutchuenensis*; C, D, *T*. *smileya* [47]; E, F, *T*. *ehrenswaerdii* [45]; G, H, *T*. *polaris* [28]; I, J, *T*. *nipponica* [47–49]; K, L, *T*. *occidentalis* [50]. Scale bars = 2 mm.

Reliable fossil records of *Thuja* with cones are limited (only 4 species until now): *T*. *polaris* was found in the Mid Paleocene sediments of the Eureka Sound Group on Ellesmere Island (Canadian Arctic) [28]; *T*. *ehrenswaerdii* (Heer) Heer from the Paleocene sediments of Kongsfjorden in Spitzbergen [45]; *T*. *nipponica* Tanai et Onoe from both middle Miocene sediments in Sikhote-Alin of Far East Russia and late Miocene Akita Prefecture in Honshu, Japan [47–49]; and *T*. *occidentalis* from Pliocene-Pleistocene sediments in Peary Land, Greenland [50]. Here we exclude a late Cretaceous record of *T*. *smileya* with cone (Fig 4C and 4D) [46], for it has several lateral seed cones arranged helically (Fig 4D), and oblanceolate or obovate bract-scale complexes [46], characters which place it outside *Thuja* [34].

**Fig 5:**
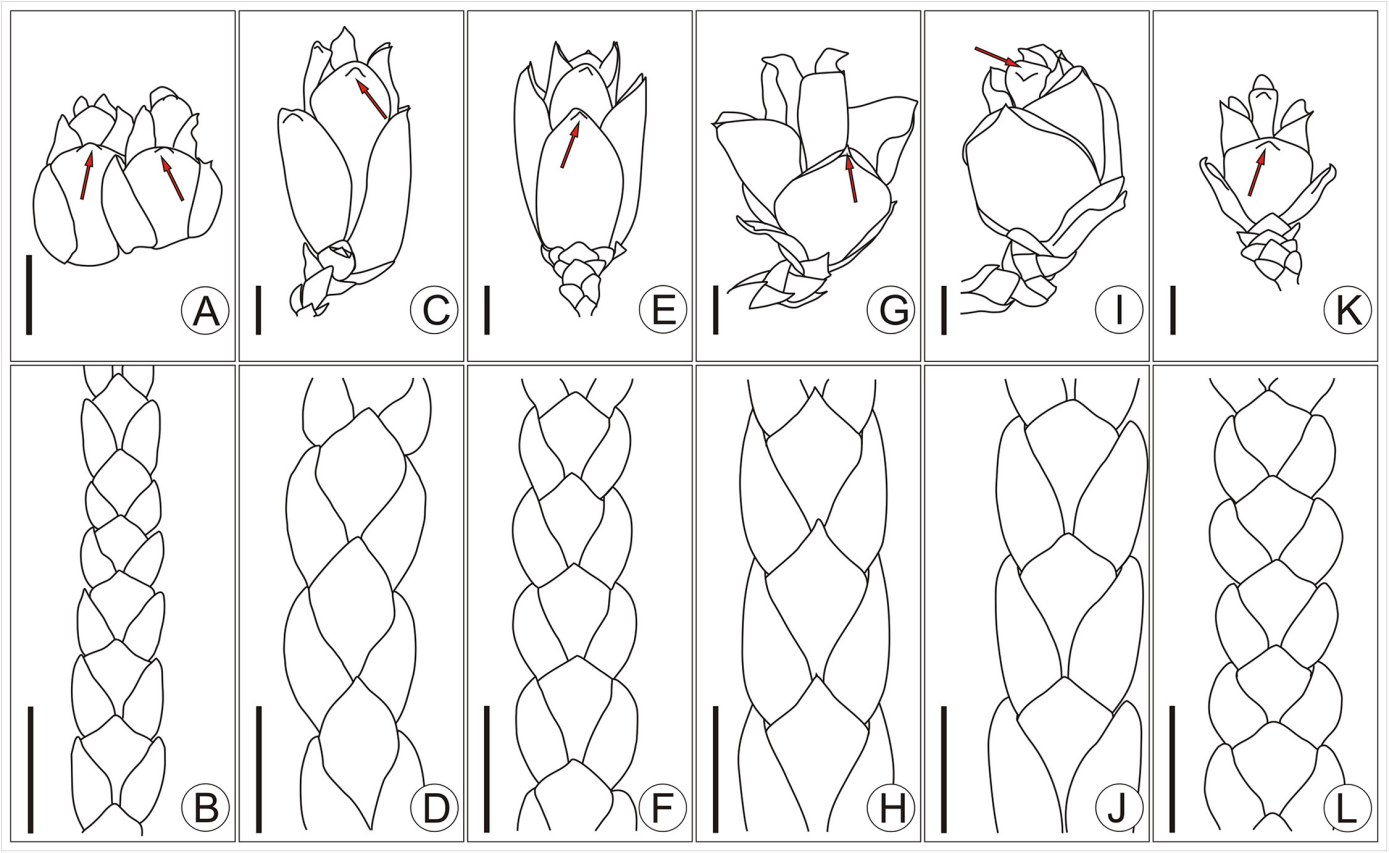
Comparison of fossil *T*. *sutchuenensis* with all 5 living *Thuja*. A, B, fossil *T*. *sutchuenensis* from this article; C, D, *T*. *koraiensis*; E, F, *T*. *occidentalis*; G, H, *T*. *plicata*; I, J, *T*. *standishii*; K, L, *T*. *sutchuenensis*.—Arrows showing the umbo; Scale bars = 2 mm (C–L are redrawn from Ref. 34).

The earliest unambiguous *Thuja* fossil is *T*. *polaris* from the Paleocene of Ellesmere Island in Canada (Fig 4G and 4H) which most closely resembles the modern species *T*. *plicata* (Fig 5G and 5H) from western North America in its morphology. However, female cones of *T*. *polaris* have more bract-scale complexes (8–9 pairs) than *T*. *plicata*, and nearly twice as many as the fossil from Zhangcun. The seed cones of *T*. *ehrenswaerdii* (Fig 4E and 4F) are small, ca. 4 mm long, with 4 pairs of decussate bract-scale complexes, lacking an umbo on the outer side of the bract, while a small umbo occurs on each bract in the fossil from Zhangcun. Based on the description of Huzioka & Uemura [49], *T*. *nipponica* (Fig 4I and 4J) is similar to the living species *T*. *standishii* (Fig 5I and 5J), i.e. the female cones are oblong, the bract-scale complexes consist of 3–4 pairs, while the basal pair of bracts is longer than the second basal pair. However, in our Zhangcun fossil, the basal pair of bract-scale complexes is smaller than the second pair, which is the biggest pair. Bennike found ovoid seed cones of *T*. *occidentalis* (Fig 4K and 4L) in the Pleistocene of Greenland. These cones were 6–7 mm long, with 4 pairs of bract-scales, only the middle ones of which were fertile. The branchlets were flattened, with decussate scale leaves, and obvious glands on the facial leaves; on the other hand, the cones from Zhangcun are only 4 mm long and the facial leaves lack glands.

It follows that there are significant differences between the Zhangcun fossil and all other fossil species of *Thuja* (Key 1).

### Key 1. Key to fossil species of *Thuja* based on cone characters

1. Bract-scale complexes 8–9 pairs...................................................... *T*. *polaris*
  Bract-scale complexes 4–5 pairs...................................................... 2
2. Seed cones ellipsoidal...........................................................................*T*. *nipponica*
  Seed cones ovoid.............................................................................. 3
3. Bract-scale complexes elliptic......................................................... *T*. *occidentalis*
  Bract-scale complexes ovate…........................................................ 4
4. Small umbo on the apical part of bract-scale complex......................Zhangcun fossil

No umbo on the apical part of bract-scale complex......................... *T*. *ehrenswaerdii*

### Comparison with Extant Species of *Thuja*

Using a recently published key based on seed cone characters [34], the Zhangcun fossil falls within the range of the extant *T*. *sutchuenensis* rather than any other living species (Fig 3; See more detailed comparison in Table 1). Besides, the cuticle characters of the Zhangcun fossil are also the same as those of *T*. *sutchuenensis* (Fig 3, Table 1) [34].

**Table 1:** Morphological characters of the Zhangcun fossil *Thuja* and the five living species of *Thuja*.

	Zhangcun fossil	*T*. *sutchuenensis*	*T*. *koraiensis*	*T*. *occidentalis*	*T*. *plicata*	*T*. *standishii*
**Female cone**						
Seed cone shape	ovate	ovate or elliptic	narrowly ovate	narrowly ovate	ovate	broadly ovate
Seed cone length (mm)	ca. 4	5–8	7–11	8–12	10–16 (–18)	7–12 (–14)
Seed cone width (mm)	ca. 3	3–4	6–9	4–6	6–8	6–7
Bract-scale complexes number	8–10	8–10	8–12	(6–) 8 (–10)	8–12 (–14)	8–10 (–12)
Bract-scale complexes shape	broadly obovate	broadly obovate	elliptic*	narrowly obovate	rhombic*	rhombic*
Widest position of the Bract-scale complexes	distally	distally	middle*	distally	middle*	middle*
Bract-scale complexes length (mm)	2.5~3.0	1.3	2.1	2.1	2.1	1.6
Bract-scale complexes width (mm)	1.0~1.8	1.1	1.0	1.1	1.3	1.6
Bract-scale complexes length/width	1.9:1	1.2:1	2:1	2:1	1.6:1	1:1
Bract-scale complexes apex	rounded	rounded	obtuse*	obtuse*	obtuse*	obtuse*
Umbo apex	obtuse	obtuse	obtuse	acute*	acuminate*	acute*
**Leaf**						
Facial leaves apex	obtuse	obtuse, serrate	obtuse, micro teeth	obtuse, no tooth	acuminate, no tooth*	acute, no tooth*
Lateral leaves apex	inturned	inturned	inturned	inturned	straight*	inturned
Overlaping between the adjacent lateral leaves	no	no	no	no	yes*	yes*
**Epidermal characters**						
Anticlinal wall of subsidiary cell	low	low	low	low	low	high*
Margin of subsidiary cell	jagged	jagged	jagged	jagged	jagged	Serrate*
Ornamentation on inner surface of subsidiary cell	coarse particles	coarse particles	smooth*	coarse particles	smooth*	smooth*
Age	Pliocene	extant	extant	extant	extant	extant
Reference	This paper	Sun et al., 2015	Sun et al., 2015	Sun et al., 2015	Sun et al., 2015	Sun et al., 2015

Note

* indicating character differences between Zhangcun fossil and other living species of *Thuja*.

### Key 2. Key to extant species of *Thuja* related to the Zhangcun fossil based on cone characters

1. Bract-scale complexes obovate, widest distally...............................................2
  Bract-scale complexes elliptic or rhombic, widest in the middle......................3
2. Bract-scale complexes narrowly obovate, apex obtuse, base acute.................................................................................................................... *T*. *occidentalis*
  Bract-scale complexes broadly obovate, apex rounded, base cuneate……...........................................................................*T*. *sutchuenensis* & Zhangcun fossil
3. Bract-scale complexes rhombic, length/width about 1:1................... *T*. *standishii*
  Bract-scale complexes elliptic, length/width more than 1.5:1.............4
4. Bract-scale complexes’ base acute, umbo apex obtuse……............. *T*. *koraiensis*
  Bract-scale complexes’ base cuneate, umbo apex acuminate............*T*. *plicata*

The above detailed comparison with fossils (Key 1) and extant species of *Thuja* (Key 2, Table 1) indicates that the fossils here should be assigned to *T*. *sutchuenenesis* because of their resemblance to the extant species.

## Discussion

**Fig 6:**
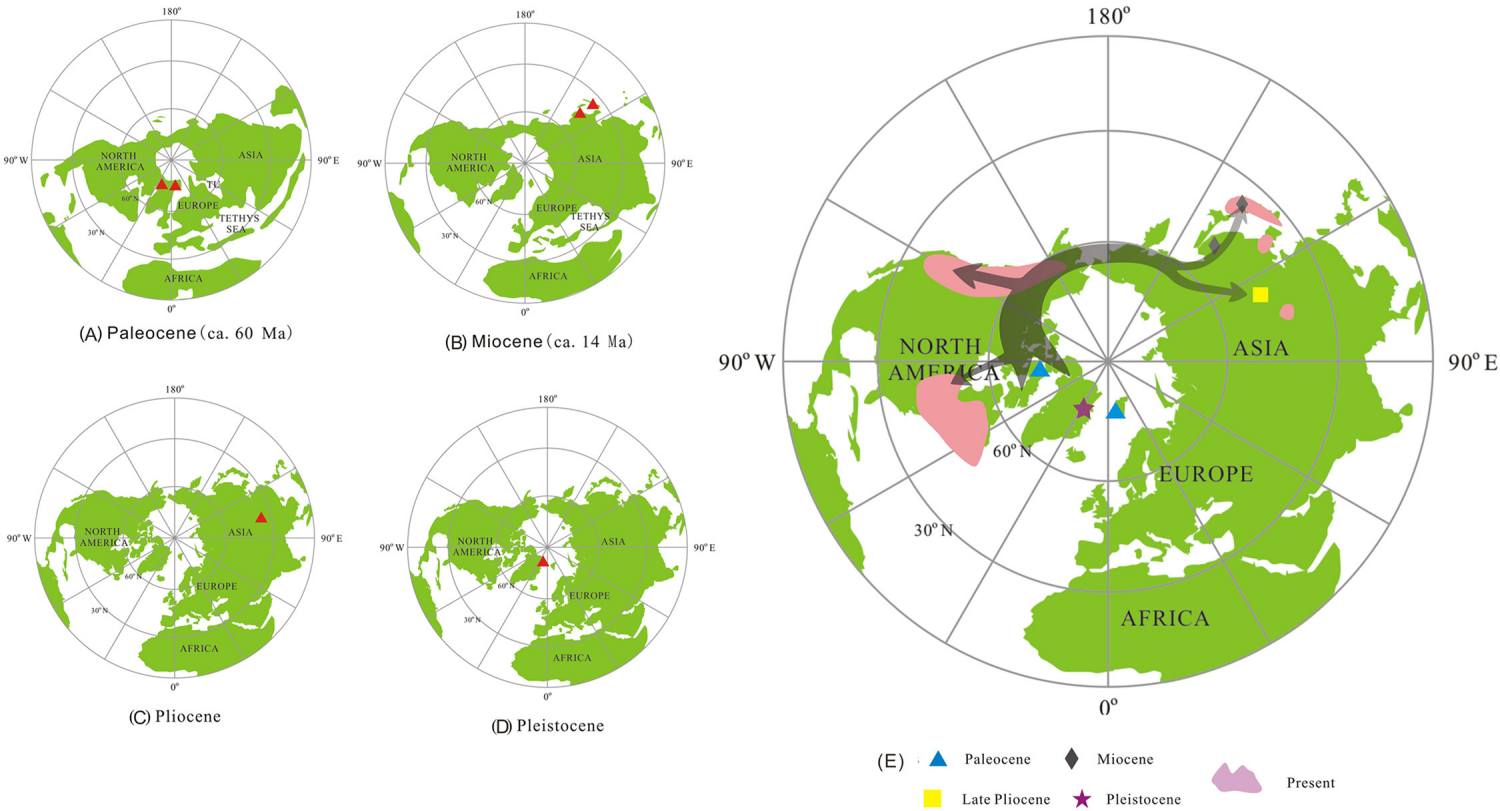
Maps showing the distribution of fossil and living *Thuja*. (A—D)▲: Fossil record of *Thuja*, projections of Lambert Equal-Area Azimuthal (North Pole); (E) Potential migration route of *Thuja*: Pink part shows the present distributional range of this genus in the world. The dark gray arrows show the possible migration route of *Thuja*. The data of palaeolatitude and palaeolongitude were converted from those of latitude and longitude of fossil sites using “PointTracker for Windows” software, and plotted on 4 individual palaeogeographical maps and 1 modern geographical map on Projections of Lambert Equal-Area Azimuthal (North Pole) by using ArcView GIS 3.2 software.

*Thuja* fossils have been widely found in sediments of Paleocene to Pleistocene age in the Northern Hemisphere from 36.8°N to 86.3°N [34–45] (Fig 6).

In the Paleocene as the Atlantic Ocean gradually expanded, North America, Greenland and Eurasia remained connected at high latitudes. However, the Turgai Straits separated Europe from Asia. The gradual disappearance of the Mid-Continental Seaway resulted in the reunion of eastern and western parts of North America, while eastern Asia and western North America were linked by a continental connection (Fig 6-B; see Map 066 in [33] and [51]). *T*. *polaris* was found in the Mid Paleocene Eureka Sound Group of Ellesmere Island (Canadian Arctic), while *T*. *ehrenswaerdii* came from the Paleocene sediments of Kongsfjorden in Spitzbergen. In *T*. *polaris* the number of scales is 16–18, roughly twice the number of *T*. *ehrenswaerdii* (8). Moreover, the foliage characters of *T*. *ehrenswaerdii* are similar to those of *Chamaecyparis*. These differences indicate that we are dealing with two distinct species. As the earliest reliable fossil record of *Thuja*–*T*. *polaris* was found in the Canadian Arctic–it seems likely that *Thuja* originated at high latitudes in western North America.

In the Miocene, as the Atlantic underwent further expansion, North America and Greenland remained united. Moreover, western North America and East Asia still had a land connection (Fig 6-C). In Asia Japan was still united with the eastern part of the Asian mainland. During this time *T*. *nipponica* was found in both Akita County (NE Honshu), and in the Sikhote-Alin area of Far Eastern Russia.

In late Pliocene the global tectonic plates had nearly the same arrangement as today. It was at this time that *T*. *sutchuenensis* grew in Shanxi Province, China (Fig 6-D). In the late Pliocene to Pleistocene sediments belonging to the Kap Kobenhavn Formation in Peary Land, northern Greenland cone bearing material of *T*. *occidentalis* has been found.

Based on *Thuja*’s spatial and temporal distribution we can speculate that *Thuja* originated in or before the Paleocene in the high latitudes of western North America. From there the conifer diffused in easterly and westerly directions (Fig 6). By the Paleocene *Thuja* had already expanded into eastern North America and Greenland. In the Miocene *Thuja* spread to eastern Asia, while the middle Miocene land connection between East Asia and western North America existed. In the late Pliocene of eastern China *Thuja* was still present. With the cooling and drying during the Quaternary *Thuja* was forced southwards and became more restricted in its distribution. In eastern Asia it was only able to survive in three confined areas, i.e., *T*. *koraiensis* in the Korean Peninsula and Changbai mountain area of China, *T*. *standishii* in Honshu and Shioku, Japan and *T*. *sutchuenensis* in the Daba Mountains, Chongqing, China [31, 52]. In North America the high latitude populations migrated southwards to mid-latitudinal areas.

In *Thuja*’s long geological history no reliable record has been found in Europe or western Asia. This indicates that we can eliminate a possible pathway through Europe into Asia. Our study supports the hypothesis of an origin in the high latitudes of North America [24, 29] and presents additional information regarding *Thuja*’s migration in time and space.
